# On Disorders of the Mind in Lying-In Women

**Published:** 1829-10-01

**Authors:** 


1. On Disorders of the Mind in Lying-in Women. By *Robert Gooch*, M.D.
2. On Puerperal Mania. By Dr. *Marshall Hall.*

It will be remembered that so long ago as the year 1819, Dr. Gooch published a paper on puerperal insanity, in the last volume of the College of Physicians. The lapse of ten years has afforded the author great additional experience; and although he does not find many errors to correct, yet he observes some obscurities that require elucidation—some opinions that deserve to be enforced—much information that ought to be communicated. He has now, therefore, "written almost a new paper on the subject," retaining out of the former only the cases, and those views which he still entertains, and finds himself unable to state more explicitly. We shall endeavour, for the benefit of those who do not possess the volume under review, to condense the more prominent features of this paper, hoping that this abridgment will induce many to peruse the original.

During the progress of pregnancy, there is no time at which the mind of the mother may not become disordered; but there are two periods more particularly obnoxious to this distressing malady—parturition (or rather a little afterwards)—and lactation. Our author has repeatedly seen the commencement of mania and of melancholia in women who were in child-bed, or who had recovered from their delivery, and were nursing. Dr. Gooch prefers relating a case or specimen of each, to a general description of the disease—and for this reason.

"Such descriptions are commonly formed of a bewildering multiplicity of circumstances which never occurred together in one and the same instance, so that they are pictures which resemble nothing in nature, like the abstract ideas of the old metaphysicians." 110.

If this maxim come to be acted on in Medical Literature, there must be a total revolution in the construction of systematic works. They must, hereafter, consist of *cases*, and not of general descriptions of diseases. It is very true that *all* the symptoms enumerated by systematic writers, *very seldom* occur in any one case—nor do we believe that the most ignorant student or wrong-headed practitioner ever expects such a thing. But as some of the symptoms *always* occur, and most of them are *generally* present, it is surely better to include all of the phenomena in a description, than merely exhibit a *specimen*, in which some, or perhaps many of the usual symptoms may be absent. We think, therefore, that Dr. Gooch's reflection (for surely it is a reflection) on systematic descriptions of disease, is both ill-timed and injudicious. We are already inundated with cases, and this plan of Dr. Gooch's would only increase the "bewildering multiplicity."

In the cases which had occurred during lactation, or rather after weaning, the author does not attribute the disease to the cessation of suckling. The symptoms in all the instances, had commenced *before* the child was taken from the breast. Nearly all these were cases of melancholia rather than mania, and there was this peculiarity about the commencement of the disease, which Dr. G. has never or seldom noticed at the commencement of mania—namely, an incipient stage, "in which the mind was wrong, yet right enough to recognise that it was wrong." We shall extract the following case, which is offered in illustration.

"A pale delicate lady, nursing an infant four months old, told me that she scarcely knew what was the matter with her: her sight was so impaired that she could not read; her powers of attention were so much impaired that her household accounts were burthensome to her; that she often rang for the footman, and when he came she had forgotten what she had rang for. She said she had a good husband, sweet children, ample property, everything to make her happy, yet she felt no interest in life. She added, that if this went on thus she should lose her senses. She had lost flesh, and had little milk. After a short time she took it into her head that she had a fatal disease, and I was called out of my bed several nights to see her die. She told me that I was quite mistaken about her case; that she was sure she was dying, and that if I would sit down for five minutes I should see her expire. She next began to accuse her friends, especially her husband, whom she charged with infidelity, and an intention to poison her; and it became necessary to separate her from her family, and place her in that state of seclusion and controul usually employed under such circumstances. She continued in this state many months, but ultimately recovered, and has had a child since without a recurrence of the disease." 115.

In the above case we confess that we cannot clearly see the proof of the patient's recognizing the wrong state of her own mind. The apprehension of losing her senses *if* the impaired condition of her sight, her attention, and her memory *continued*, is certainly no recognition nor profession of wrong mind—if by wrong mind Dr. G. means mental derangement. We conceive, also, that the above case was one of mania rather than melancholia. The ideas that her husband was unfaithful, and that her friends were attempting to poison her, were as insane ideas as any that emanate from an inmate of Bedlam. Neither was the process of cure that which would be proper or successful in melancholia—" seclusion and control." We do not suppose, however, that Dr. G. intends this case to represent one of melancholia, although the fear of dying certainly belongs to the disease. Our author remarks that if a physician were taken into the chamber of a patient whose mind had become disordered from lying-in or nursing, he would not be able to tell, by the mere condition of her mind, that the disease had originated in such causes. In some cases the phenomena bear a considerable analogy to those of delirium tremens—in others, there are cataleptic symptoms. A curious case of this last kind is introduced by our author, but is one of those previously published.

In giving an opinion as to the probable result of puerperal mania, there are two questions—is there danger of life?—how long will the disease last; and what chance is there of its becoming permanent? Dr. G. remembers the time when it was the prevalent opinion among eminent physicians, that puerperal mania was never fatal.

"Whilst I was attending the near relation of one of the most eminent and experienced of the provincial practitioners of this island, a letter arrived from him, begging the family to have no fears, that he had seen many such cases during his long life, and never saw one die; and even the late Dr. Baillie, when consulted about a case, remarked, 'that the question was not whether she was to get well, but when she was to get well.' The latter patient died within a week after this prognosis. There can be no doubt that a very large proportion of cases of disordered mind in lying-in women and nurses ultimately recover, but it is equally certain that some of them die, and there are two modes of calculating the probability of death in any individual case; the one is to ascertain the proportion of deaths to recoveries in as large a number of cases as possible, the other is to endeavour to discover some symptoms, the absence or presence of which indicates safety or danger." 121.

As to the former of these modes, it is very difficult, we should say almost impossible, to procure trust-worthy information. M. Esquirol has made the proportion 6 deaths in 92 cases:—Dr. Burrows, 10 in 57. But to say that the probable mortality is 1 in 15, or in any other number, might do very well for an actuary at an Insurance Office—it would be a ridiculous answer in the mouth of a medical practitioner. An interesting question then presents itself—" are there no symptoms in this as in other diseases, by which to judge whether or no the life of the patient is in danger?" The following passage from the manuscript Lectures of Dr. William Hunter, is considered very valuable by Dr. Gooch.

"'Mania,' says Dr. Hunter, 'is not an uncommon appearance in the course of the month, but of that species from which they generally recover. *When out of their senses, attended with fever like paraphrenitis, they will in all probability die*, but when without fever it is not fatal, though it (i. e. fever) generally takes place before they get well. I have had several private patients, and have been called in where a great number of stimulating medicines and blisters have been administered, but they have gone on as at another time, talking nonsense till the disease has gone off, and they have become sensible. It is a species of madness they generally recover from, but I know of nothing of any singular service in it." 123.

Making allowance for loose language and probably inaccuracy in the notes of extemporaneous language, and comparing Dr. Hunter's statement with his own experience, Dr. Gooch would extract from the above passage the following meaning:—

"That there are two forms of puerperal mania, the one attended by fever, or at least the most important part of it, a rapid pulse; the other accompanied by a very moderate disturbance of the circulation; that the latter cases, which are by far the most numerous, recover; that the former generally die. This agrees closely with my own experience. Cases 8, 9, 10, which terminated fatally, were all attended by a very rapid pulse. None of those with a slow, or only moderately excited pulse died. Some which were attended with a quick pulse recovered; but none of these were treated for paraphrenitis." 123.

There are some other circumstances to be taken into the account of prognosis; viz. the form of the derangement, and the period at which it occurs. Mania soon after delivery is more dangerous to life than melancholia beginning several months afterwards. Nights passed in sleep, a pulse slower and firmer, even although the mind continues disordered, promise safety to life. On the contrary, incessant pervigilium, a quick, weak, fluttering pulse, and all the symptoms of increasing exhaustion, portend a fatal termination, even although the condition of the mind may be apparently improved. In the fatal cases, the patients have died with the symptoms of exhaustion, not those of oppressed brain, with a single exception.

But supposing the patient lives, how long is the disease to last? What danger is there of its permanency? Mania is less durable than melancholia —it is more dangerous to life—less dangerous to reason. The records of hospital practice afford us no data as to the probable duration of puerperal mania. Hospital cases are picked obstinate cases. Those which last only a few days or weeks, (which form a large proportion) are totally lost in the estimate of a Lunatic hospital. The tables of Esquirol, Burrows, Haslam, &c. "throw little light upon the question, and present a prospect unnecessarily gloomy and discouraging." Of the many patients about whom our author has been consulted, he knows only of two who are still, after many years, disordered in mind—and of these, one had been deranged before marriage. In respect to the repetitions of the disease after subsequent pregnancies Dr. G. thinks there is very little predisposition left—but still a sufficient possibility of such an event to call for the utmost degree of care in all subsequent confinements.

We now come to the etiology of puerperal mania, as well as its essence or pathology. Leaving aside all discussion respecting "*that peculiar state of the sexual system which occurs after delivery*,"—and which we cannot satisfactorily explain, although we may be convinced of its existence as a predisposing cause of the disease in question, we are informed by our author that a large proportion of the cases (as far at least as his experience goes) have occurred in patients, in whose families disordered mind had already appeared.

"Among the causes of this disease, there are two others which require notice—the one a disordered state of the organs of digestion, the other weaning. As to the first, it was very manifest in some cases, in others less so. Mental derangement is said to be often produced by weaning, that is, by the sudden suppression of the secretion of milk. I have no right to deny the experience of others, but my own would never have led me to such a conclusion. Among the fashionable women of this town nothing is so common as not to nurse their children; the milk comes in about one or two days after delivery, and the breasts become as hard as stones, but not a drop is extracted; and sometimes by cold spirit lotions constantly applied to the breasts, sometimes by embrocations of oil and brandy, sometimes by poultices (according to the whim of the nurse, the patient, or the medical attendant,) with gentle aperients, the milk is suppressed in a few days. I must have known this done in more than a hundred instances during the first week after delivery, a time much more liable to disordered mind than a later period, and in not one did it occasion puerperal insanity. In all the cases which I have seen months after delivery, the weaning has been the consequence of the disease, not the disease the consequence of the weaning. The patients had been reduced in health by nursing, their memories had become enfeebled, their spirits depressed, and their minds ultimately disordered; and they were directed to wean their children because they had neither milk nor strength to enable them to nurse." 130.

We shall find that this etiology is very strongly supported by Dr. Marshall Hall, from whose book we shall extract the following passage.

"There is a mixed case which shows itself under a still different form, from any which have hitherto been described:—it is *puerperal mania*. I believe this disease to result, in general, from all the circumstances following parturition combined; but chiefly from the united influences of intestinal irritation and loss of blood. I purpose to pursue this subject hereafter. In the mean time, however, I would observe, that I am persuaded that real puerperal phrenitis is comparatively a rare disease,—that puerperal mania is seldom of an inflammatory character, and that it is, especially, to be treated by those measures which are suited to the mixed case of intestinal irritation and exhaustion. This opinion is confirmed by the fact of mania occurring from undue lactation, as well as from the circumstances of the puerperal state. I am inclined to attribute much more to the combined influence of irritation and exhaustion, than to the mere 'state of the sexual system which occurs after delivery,' which has been assigned as the chief cause of this morbid affection by Dr. Gooch, in a most interesting paper upon this subject, in the sixth volume of the Transactions of the College of Physicians, p. 280,—although I would by no means exclude the influence of this principle altogether. There is ample evidence, in Dr. Gooch's cases, of the influence of intestinal disorder; and the events of labour, and the circumstances of lactation, ever add to this a state of exhaustion. This view is the more important, because it directly suggests the proper mode of treatment, which consists in restoring the system to a state of due health by every means in our power, whilst we adopt every measure which can soothe and allay the morbid irritability of the nervous system.

"I am confirmed in this view of the nature of puerperal mania, not only by a careful investigation of its causes, and the good effects of the remedies which I have mentioned, but by having met with the symptoms of intestinal irritation described in chapter V., as a prelude to those of mania." 253.

From the foregoing passage it will be evident that Dr. Hall does not exclude the peculiar condition of child-bearing from among the causes of puerperal mania, though he endeavours to trace the disease, in many cases at least, to more tangible and explicable causes, namely, debilitants and depletives during or after delivery. This, indeed, is the etiology, as far as we can understand it, which Dr. Gooch himself employs.

We next come to the proximate cause—"the morbid state of organization on which the disorder of the mind depends." Dr. Gooch very properly stigmatises the popular and professional disposition to attribute "raving of the mind to inflammation of the brain." Nothing can be more dangerous than such a doctrine, even in common, and, à fortiori, in puerperal mania.

"But experience and reflection lead to very different conclusions; they teach us that a disorder of the mind may be connected with very opposite states of the circulation, sometimes with inflammation or active congestion, for which depletion is the shortest and surest remedy; sometimes with an opposite condition of the circulation, which depletion will only aggravate.

"Cerebral excitement does not necessarily depend on inflammation or congestion, nor is depletion, however moderate, necessarily the proper remedy. Cerebral excitement is often aggravated by depletion; and in some cases, as I shall have occasion to relate, absolutely brought on by it. Now the question, what is the morbid state of organization on which puerperal insanity depends, must be determined in the usual way. There is only one safe mode of working the problem, by observing the causes which brought on the disease, the bodily symptoms which accompany it, the way in which it is affected by remedies, and the morbid appearances discovered after death." 132.

We shall glance at some of the cases here, in order to render this article as complete as possible.

*Case.* A lady became deranged a few days after labour, attended by an alarming hæmorrhage. When Dr. G. saw her, she was sitting up in a chair, looking from side to side, but unwilling or unable to answer questions. Her face and lips were colourless—pulse small and quick. Nothing was done but regulating the bowels, and she recovered.

*Case.* A lady was delivered easily, but the placenta adhered, and was separated by the hand. The after-pains were severe, and at length settled into permanent pain and tenderness in the hypogastrium, with a sharp pulse of 116. The lochia ceased—she had slight rigors. An eminent physician considered it hysteritis, and for three days, blood-letting, both general and local, were freely employed. The pain and tenderness continued; but the patient was now so much reduced, that the lancet was suspended, and a consultation called. The result was a determination to bleed *usque ad deliquium.* The first cups were buffed, the others were not. Five were taken away before faintness was induced. At 4 o'clock next morning the nurse waked Dr. G. saying her mistress was "much changed," and she thought her dying. He found her cold, clammy, with thready pulse, and pale sharp features. Her mind rambled. Warm wine and water was given in small but frequent doses, and next morning she was much recruited. The pain and tenderness of the uterus were gone. In the evening she was maniacal, with pulse 140. Some more wine was given, and she became calmer under its influence. Next morning puerperal mania was evident to all the medical attendants. In this state she continued some weeks, during which it was necessary to put on the straight-waistcoat. In a month she was convalescent from mania; but her abdomen swelled, and she died dropsical in eleven weeks after her confinement.

Dr. Gooch might have added, without much violation of probability, that the doctors were good friends to the undertaker in this case. They cured equivocal hysteritis—but they laid the foundation for the dropsy.

*Case.* Dr. Gooch went to see a lady who had become maniacal on the 7th day after delivery, and after unusually low living in consequence of some little fever. Dr. G. found her lying on one side, apparently engaged in deep thought, and quite inattentive to surrounding objects. She was in a profuse perspiration, pulse 140 and weak. She had spasmodic twitchings and picked the bed-clothes. Dr. G. was, at first, a little doubtful of the nature of the disease. It had some resemblance to delirium tremens. In consultation it was determined to give 30 drops of Battley in two doses. Next morning Dr. G. was met with cheerful countenances, and the agreeable intelligence that the patient was "quite well." The second draught had thrown her into a sound sleep, from which she awoke with a calm clear mind, and a pulse of 80. We agree with Dr. G. that—"depletion would easily have converted this into a dangerous and perhaps fatal case." The following case is particularly instructive.

*Case.* "Mrs. —— was delivered of her first child after a natural labour, attended, however, by rather more than the usual loss of blood. From the first her manner appears to have been excited and unnatural. Her nights became restless, her mind more excited, and about three weeks after her delivery she became maniacal. Her pulse was 140 before any active remedy was employed. She was put upon low diet, leeches were applied to the head, and she was freely purged with calomel and castor oil. The symptoms not abating, and the patient becoming very violent, a cupper was sent for, who took ten ounces of blood from the head by cupping glasses, and the following morning I saw her for the first time. She was sitting up in bed in a straight-waistcoat. Whatever was asked her she did not answer, but repeated it like an echo, 'Have you any head-ache?'—'Have you any head-ache?' 'Put out your tongue'—'Put out your tongue'—she would not say any thing else. Her tongue was moist and pale—her pulse was between 120 and 130, small and weak—her bowels had been lately freely moved—her skin was not hot, her face was very pale—she had had no sleep for many nights. This being the state of things, I thought it a great object to procure repose. She therefore took twenty minims of the sedative solution of opium, and ten minims in a two ounce glyster every six hours. This procured six hours of uninterrupted sleep, and when awake she was more herself. Some symptoms led her attendant to employ another bleeding by ten leeches to the head, which was shaved, and a blister applied to the crown. When I saw her two days after my first visit, she had had in the night several hours of sleep, and was so much better in mind, that her friends were surprised when I told them that she was not out of danger. Her face was very pale, and her pulse so quick, small, feeble, and fluttering, that I remarked to her medical attendants that she would not bear the loss of another ounce of blood. It was agreed that she should continue her small opiate glysters, and that care should be taken to supply her with sufficient nutriment; but the next day the symptoms of exhaustion became more alarming, and when one of her medical attendants visited her in the evening, he found her pale, cold, breathing only at long intervals, and with scarcely any pulse—she died that night. The body was opened the next day by two very experienced anatomists. The veins throughout the body were remarkably empty—the heart contained little blood—the lungs and liver were singularly pale. Within the head there was the same deficiency of blood in the veins of the pia mater, and in the sinuses—under the arachnoid membrane was a little serum. On slicing off the hemispheres the bloody points were unusually numerous." 140.

No man of the least reflection can hesitate to ascribe the death of this patient to the depletive measures which were employed—insanely employed!

*Case.* Our author accidentally saw a female who was sitting up in bed in a straight-waistcoat, with a flushed cheek, a dull eye, and occasionally uttering unintelligible words. Her pulse was above 100. She was blooded that evening to faintness, which did not require more than eight ounces, and died in a few hours afterwards. On examination, the viscera of the abdomen were found to be healthy, as was also the peritoneum. The internal and external surfaces of the uterus appeared natural. There was about half a pint of reddish serum in the cavity of the abdomen.

The following inferences which Dr. Gooch draws from the consideration of eleven cases detailed in the volume, (out of which we have selected the above,) we shall lay before our readers in the author's own words.

"Let the reader reflect on the leading points of these cases. In No. I. the disease occurred in a pale lady, without any heat of skin, or much quickness of pulse, and was not relieved by the loss of blood. In No. III. it occurred in one whose constitution was drained and enfeebled by nursing. In No. IV. it occurred in a pale woman, habitually hysterical, subject to bear dead children from want of power to afford them life for nine months. In No. V. it occurred in one who had been drained by flooding. In No. VI. in one in whom, for urgent reasons, the circulation had been reduced to the lowest ebb consistent with life. In No. VII. in one who had been living very low for a week, with such marked symptoms of the irritation of debility that at first sight I thought it was the close of some disease that had been overlooked. It was speedily relieved, not by cupping and purging, but by the tranquillizing and sustaining power of opium. In No. VIII. the disease was treated, though with all possible prudence and moderation, as an inflammatory state of the brain, by leeches, cupping, purging, and low diet, yet the patient died, not with symptoms of oppressed brain, but with those of exhaustion; and on examining the body the whole venous system was found extraordinarily empty of blood. In No. X. the patient fell, as if shot, under the stroke of the lancet, and on examining the head there was found no effusion, and empty blood vessels. In No. XI. the disease came on after puerperal convulsions, a disease generally but not always depending on cerebral congestion, and after one of those enormous bleedings commonly practised in these cases, and no morbid appearances were discovered after death in the brain.

"These cases, if fair specimens of puerperal insanity, lead straight to the conclusion, that the disease is not one of congestion or inflammation, but one of excitement without power; I shall be asked, are not these picked cases, selected to prove a point, and forming a small proportion to those of another character? Their very number gives the negative to this suspicion: ten cases can never form a small proportion of the experience of one individual, however extensive his opportunities of seeing the disease may be; for puerperal insanity is not like fever, a disease in which an experienced physician counts his cases by hundreds. Dr. Wm. Hunter said, that in the course of his practice he had met with about twenty or thirty. There can be no mistake, unless by some extraordinary accident, all my cases have been exceptions to the general rule—an incredible supposition! It is true I have related those in which the nature of the disease was most distinctly marked, in which the truths I am endeavouring to explain were most legible; but in most of the remainder there was nothing to contradict these conclusions. It was the same form of disease, only less marked and striking. They surely prove that those cases of puerperal mania which are attended by a very rapid pulse, which Dr. Wm. Hunter said generally die, and which he attributed to paraphrenitis, do not depend on this state of the brain, which requires depletion, but on a more exhausting excitation of the nervous system, which requires soothing and sustaining treatment." 147.

It would be ridiculous to conclude that a few bloody points in the cerebral substance, and a little serum under the tunica arachnoidea, (in one of the cases,) should authorise us to treat this disease as inflammation or congestion of the brain, when the symptoms, the mode of death by exhaustion, and the remarkable emptiness of the veins after death, gave the negative to the assumption. Dr. Gooch makes many judicious reflections on the use and abuse of post-mortem examinations. Morbid anatomy, to prove instructive, must be attended by two requisites.

"An eye familiar with the difference between natural and morbid appearances, and a mind capable of interpreting the hieroglyphic characters left by disease. These qualifications are never found, except in those who are, or at least have been, for a considerable portion of their lives continually employed in these examinations. A man whose experience in morbid anatomy amounts to five or six examinations in the year, is neither a competent witness of appearances, nor a competent judge of their meaning. To understand what these appearances mean, it is necessary to know the history of the case during life, the symptoms by which it was attended, and the way in which it was affected by remedies. Those pathologists who consider increased vascularity of the brain, and an effusion of fluid, however slight, as infallible signs that congestion or inflammation existed during life, and that depletion was the essential remedy, will do well to read Dr. Kelly's Paper on the Pathology of the Brain, and Dr. P. M. Latham's Account of the Epidemic at the Milbank Penitentiary." 150.

To the question, is there no such disease as phrenitis in parturient women? Dr. G. answers that, according to his own experience, phrenitis, that is, furious delirium from inflammation of the brain, is a rare disease in childbed. Inflammatory head-aches, attended with delirium, are occasionally met with, but these are very different from puerperal mania.

The 13th case in the work before us is interesting, as shewing the extensive influence of morbid secretions on the organ of the mind—and indeed on the mind itself.

A sensitive female became maniacal a few days after her confinement, and a straight-waistcoat was necessary. Dr. G. found her with hot skin, full pulse, about 100—dark furred tongue—confined bowels—stools dark and offensive. Small doses of calomel and jalap produced but scanty motions, and no relief. She talked incessantly, and never slept. She was very violent. After three days some powerful aperients were given, which procured a very large evacuation, "nearly black, and horribly offensive," being as usual discharged into the bed without notice.

"It acted again an hour or two afterwards, but now the nurse, who was sitting by the bed-side, was surprised to see her turn round, and in a calm and natural manner request to be taken up as her medicine was going to operate; her waistcoat was immediately loosened and she was taken out of bed, when she voided a stool of prodigious size, as dark and offensive as the first, and then walked back to her bed calm and collected; we saw her not many hours afterwards; her waistcoat was off, she was lying on her sofa perfectly tranquil, answered questions correctly, manifested no vestige of her complaint, excepting some strangeness in the expression of her countenance, and a timidity and abstinence from conversation which was not natural to her; she recovered rapidly and uninterruptedly." 158.

It now remains to speak of the treatment of puerperal mania. So much, however, has been disclosed in the detail of the cases themselves, that our author has little else to do than collect the rules of conduct dictated by the cases, and put them in a compact shape.

"I. The constant attendants on the patient ought to be those who will controul her effectually but mildly, who will not irritate her, and will protect her from self-injury. These tasks are seldom well performed by her own servants and relatives.

"If the disease lasts more than a few days, and threatens to be of considerable duration, her monthly nurse and own servants ought to be removed, and a nurse accustomed to the care of deranged persons placed in their stead. Such an attendant will have more controul over the patient, and be more likely to protect her from self-injury. She should never be left alone, and every thing should be carefully removed with which self-injury can be effected; such as cutting instruments, garters, handkerchiefs, towels. The windows of her chamber ought to be carefully secured. With regard to the removal of her husband and relations, this also will be a question if the disease threatens to be lasting; it is generally right. Interviews with relations and friends are commonly passed in increased emotion, remonstrance, altercation, and obviously do harm; large experience, also, is decidedly favourable to separation as a general rule, yet there may be exceptions which the intelligent practitioner will detect by observing the effect of intercourse. The husband ought never to be left alone with his deranged wife, for obvious reasons. I have known more than once a neglect of this rule produce consequences which left in the minds of those concerned a never-ending regret. On this subject a serious appeal ought to be made to the sense and feelings of the husband.

"II. The next rule regards the diet of the patient. It ought never to be very low; the lowest ought to consist of nutritious and unheating fluids, such as equal parts of gruel and milk, or gruel and good veal broth, or milk alone; and of these a quart ought to be given in the twenty-four hours. If there is any heat or thirst the broth had better be omitted; but the cases in which this diet requires to be reduced are few; it even sometimes requires to be mended. If the patient is pale, and the temperature of the skin lower than natural, it is useful to add to the above diet two ounces of wine daily, mixed with gruel. When the patient is in such a state of mind as not to ask for support, and even object to take any, a thoughtless nurse will allow hours, and even days, to pass with no other food than a cup of tea or water-gruel, at long intervals—a neglect which I have known to be of serious consequences; but if the disease after many days continues unabated, a daily portion of solid meat may be necessary, and the rule for it is this: if there is nothing in the bodily symptoms, separate from the disorder of the mind, which forbids it, this state of the mind is no objection to, but rather an argument for it. Hospital patients are sometimes clearly benefited by a cup of caudle several times a day; but to them diffusible stimulants are more safe and necessary than to persons of temperate habits. After being long accustomed to a daily supply of gin, they come into a lying-in hospital, suffer pain, lose blood, live on water-gruel, and take purgative medicines. If mania attacks them under these circumstances, a moderate quantity of wine is sometimes strikingly beneficial. Thus I would manage the diet in mania which occurs soon after delivery; but when melancholia attacks a woman long after delivery, who has been drained and enfeebled by nursing, a nutritious and even cordial diet is necessary in all cases. She should take meat every day, with about four ounces of wine. Cupping, low diet and purging would confirm her disease, and perhaps convert it into idiotism. Lastly, if mania attack a woman after sudden weaning, so that there is reason to believe that the disorder of the mind has been caused by the sudden suppression of milk, (a case very different to that which I have last described, and one which I have not witnessed,) there would be reason to suspect an inflammatory affection of the brain; but this must be determined, and the treatment regulated, not by the disorder of the mind, but by the bodily symptoms which accompany it." 161.

III. The third rule relates to medicinal treatment. The remedial agents are—bloodletting—emetics and purgatives—anodynes—tonics and stimulants. In respect to the first agent, the result of Dr. Gooch's experience is decidedly against it in mania, melancholia, and those cases which resemble delirium tremens. He does not say that cases never occur which require this remedy—but he has never met with them.

In regard to remedies for the evacuation of gastric and intestinal impurities, the activity with which they are to be employed must depend on the distinctness with which these states are present. If the powers of the constitution are not low, and the gastric symptoms are well marked, namely, a foul tongue, an offensive breath, a yellow eye, an ipecacuan emetic may be given: but where the face is pale, the skin cold, and the pulse quick and weak, the depressing influence of vomiting is to be avoided.

When the stools are unhealthy, one or two active purges ought to be given —such as the compound aloetic pill, or the compound decoction of aloes. But where the gastric symptoms are slight, and the powers of the system much exhausted, active and prolonged purgation is injurious.

### IV. SEDATIVES.

"The most valuable medicines in the treatment of puerperal mania are narcotics. If given at proper times and in proper doses, they often procure nights of better sleep, and days of greater tranquillity. This calmness is most likely to be followed by some clearing up of the disorder of the mind. These remedies produce these salutary effects much oftener in the mania of lying-in women than in mania occurring under other circumstances; for it is more uniformly a disease of nervous excitement and debility. If the head is hot, the cheek flushed, and the patient thirsty, they ought to be postponed; but if these symptoms have been removed, or are not present, sedatives ought to be given, and the most efficient, first. After many days and nights passed in perpetual wakefulness, it is an urgent object to procure tranquil sleep. For this purpose twenty minims of the sedative solution of opium may be given at once, and repeated in two hours if the patient is not asleep; even a third dose may be given in two hours more if the two first doses have failed, but the cases in which opium has been most successful have required at most two full doses. When sleep has once been procured, small doses, such as five or ten minims, should be given at intervals of six hours. If these small doses procure sleep by night it is unnecessary to return to the larger doses, but these may be used occasionally when the smaller doses fail. Constipation must be prevented by a daily dose of the compound aloetic pill or decoction, or, if these fail, by the compound extract of colocynth, which is made more soluble and active by mixing it with one-third of soap. If the sedative solution of opium should produce any of the ill effects which this drug is known occasionally to produce, such as head-ache, foul tongue, sickness, heat of skin, it should be discontinued, and the milder narcotics tried, of which the best is hyosciamus mixed with camphor; five grains of each may be given every six hours, but the night-dose should be doubled. It may be dissolved in an ounce and a half of camphor mixture. When once opiates have attained their objects they should be withdrawn, not suddenly, but gradually, diminishing the dose, lengthening the interval, watching the effect of this abstraction of the remedy, mending the diet whilst withdrawing it, and returning to the old doses if the diminution of them occasions any unfavourable symptom." 166.

V. There are cases and times when stimulants or tonics must be given. The total absence of febrile symptoms, the paleness of the face, coolness of the skin, and feebleness of pulse, will authorize the exhibition of a scruple of carbonate of ammonia, in four doses, at four-hour intervals. The time comes when opiates have been tried and are no longer necessary, or have failed. The disease threatens to last a long time, and the great object is to support the patient through a long and exhausting disease. This is best done by nourishing diet and the mineral acids, with or without bark or bitters.

VI. The last rule relates to seclusion and control.

"There can be no doubt that it is generally necessary and useful to separate the patient from all those persons who are sources of excitement of any kind. This, however, can be effected only in one of two ways—either in a separate house, or part of a house where the patient has no other associates but her nurses, or in a receptacle for the deranged, where she has no other associates than her nurses, and persons similarly afflicted with herself. This is the only society she has, excepting the short and occasional visits of the physician. Thus the power of controuling her, even by force, is placed in the hands, not of enlightened and benevolent persons, but of uneducated menials. I do not know how it can be otherwise, though I wish it could; but I think such a charge ought never to be placed in such hands without the most vigilant scrutiny of its exercise. There may be cases, or there may come a time at which some interruption to this solitary life may be advisable. When the disease has lasted long, when the patient expresses a strong wish to see some near friend, when she entertains illusions, which the sight of some one may efface, the admission of such person is worth a trial." 168.

There is no doubt that patients when recovering, or even when recovered, very often relapse, when too soon introduced to their friends. But these are not those whom our author here notices. The advice is given respecting those who have *not* recovered and are *not* recovering—who linger month after month without amendment, and whose mental delusions assume a shape accessible to moral impressions. An interesting case is related in illustration, to which the reader is referred.

Before concluding this article, we deem it but justice to state that a comparison of Dr. Gooch's Essay with the chapter on Puerperal Mania in Dr. Burrows' work, induces us to think that the former author has made free with the ideas of the latter, forgetting to acknowledge the source of the said ideas. Our closing limits prevent up from instancing many of these parallelisms:—but the following example will suffice.

Burrows. "All emotions of the mind, it is evident, are capable of disturbing the corporeal functions; and, though in themselves moral causes, they become physical in their operation."—*On Insanity, p.* 22.

Gooch. "It appears, therefore, that emotions of mind are capable of disturbing the organs of the body; and though moral causes in themselves, they may be physical in their operation." *On Puerperal Mania, p.* 187.

We do not positively assert that such parallelisms as the above (and many more might be adduced) are not purely accidental; but we can only say that we have never known such accidents in the whole of our literary life.

